# Seeing by Touch: Evaluation of a Soft Biologically-Inspired Artificial Fingertip in Real-Time Active Touch

**DOI:** 10.3390/s140202561

**Published:** 2014-02-07

**Authors:** Tareq Assaf, Calum Roke, Jonathan Rossiter, Tony Pipe, Chris Melhuish

**Affiliations:** Bristol Robotics Lab, T Block, Frenchay Campus, Coldharbour Lane, Bristol, BS16 1QY, UK; E-Mails: calum.roke@brl.ac.uk (C.R.); Jonathan.Rossiter@bristol.ac.uk (J.R.); Tony.Pipe@brl.ac.uk (T.P.); Chris.Melhuish@brl.ac.uk (C.M.)

**Keywords:** shape recognition, object features, optical-based tactile sensor, real-time processing, touch sensor

## Abstract

Effective tactile sensing for artificial platforms remains an open issue in robotics. This study investigates the performance of a soft biologically-inspired artificial fingertip in active exploration tasks. The fingertip sensor replicates the mechanisms within human skin and offers a robust solution that can be used both for tactile sensing and gripping/manipulating objects. The softness of the optical sensor's contact surface also allows safer interactions with objects. High-level tactile features such as edges are extrapolated from the sensor's output and the information is used to generate a tactile image. The work presented in this paper aims to investigate and evaluate this artificial fingertip for 2D shape reconstruction. The sensor was mounted on a robot arm to allow autonomous exploration of different objects. The sensor and a number of human participants were then tested for their abilities to track the raised perimeters of different planar objects and compared. By observing the technique and accuracy of the human subjects, simple but effective parameters were determined in order to evaluate the artificial system's performance. The results prove the capability of the sensor in such active exploration tasks, with a comparable performance to the human subjects despite it using tactile data alone whereas the human participants were also able to use proprioceptive cues.

## 1. Introduction

The sense of touch plays a particularly valuable role in physical and safe interactions, allowing the direct perception of parameters such as shape, texture, stickiness, and friction. These parameters cannot be easily attained from any other sense. As a result, either alone or in conjunction with other senses, tactile information can be used to build a perception of objects and the environment that would be otherwise unobtainable. Tactile information therefore offers a number of benefits that arise from better environment perception including the provision for safer movements and increased dexterity. Its importance for artificial and robotic systems is clear, and accordingly, there has been a rapid growth of the number of related publications since the 1980s [1,2].

However, one of the main open issues in robotics is the development of an effective sensory feedback system for robotic platforms. Such a system is targeted to achieve diverse objectives. For example, humanoid robots, surgical robots and learning robots focus on using tactile sensory feedback to increase the dexterity of the robotic arms/hands, whereas biologically-inspired approaches to humanoid robotics aim to develop an artificial platform able to interact with and understand the real world [1,3,4]. Recent technological advances combined with a deeper understanding of biological systems now make it possible to develop more versatile and sensitive sensors than were previously possible. This should benefit all robotics applications where tactile feedback is useful.

Many kinds of tactile sensor designs can be found in literature, ranging from simple [5,6] to the more complex in terms of mechanical and processing burden [7,8]. A wide range of tactile features are employed for exploring the environment, including contact detection, force measurements, force distribution vectors [9,10], strain extraction, surface traction field [11], vibration monitoring, grip force [12,13], and object recognition [7].

In this paper, the performance of an artificial fingertip sensor is investigated. This sensor has inherent safety features due to the softness of its sensing surface and an extremely diverse sensing capability that could address some of the open issues in robotics. The tactile sensor uses efficient algorithms to identify higher level features from its optical sensors. These features allow shape reconstruction by generating an image that could be then processed with image processing algorithms.

Previous work with this sensor includes: an initial sensing performance evaluation for force and 2D shape detection [14]; use in a tactile feedback system for soft object interaction, where it was employed to measure spatially-distributed skin deformation (3D shape) [15] and lateral skin displacement due to shear forces [16]; and investigation into texture discrimination, including the effect of adding a textured outer surface akin to fingerprints [17].

This paper focuses on evaluating the fingertip tactile sensor for real-time contour-following tasks in a structured environment. Such tasks have previously been shown as important for evaluating the capabilities of sensors and their processing algorithms [18,19]. Such a test is suitable for multiple reasons: (i) it is a simple but effective task, (ii) it is related to daily actions not only for object recognition but also for human interactions. In order to estimate the sensor's capabilities during these tasks, its performance has been compared to that of humans during similar tactile-based contour-following tasks. This is achieved by collecting data on the trajectory taken by the human subjects and by the artificial finger platform. These human tests do not aim to improve our understanding of the human touch capability, instead the work aims to define a robust methodology that exploits the sensor's broad real-time sensing capabilities for contour following, with a performance comparable to that of humans.

The major contributions of this work are the introduction of a suitable sensing and gripping solution, and the rapid extraction of high level features from the tactile sensor during environmental exploration and continuous active touch activities. Active touch is the act of physically exploring an object in order to learn more about it. The extracted features are highly suited to further machine learning tasks in higher level object abstraction and environment mapping applications.

This paper is structured as follows: The following two subsections describe the tactile sensor and the feature extraction algorithm; Section 2 illustrates the experiments, the set up and the methodology for the artificial exploration and the human tests; Sections 3 and 4 report and discuss the results respectively; and finally, Section 5 concludes the paper.

## 1.1. Tactile Sensor

In humans, a large proportion of the tactile information needed for object manipulation comes from the hands alone. The fingertips are consequently one of the most sensitive areas used for the recognition of object features, and have the highest density of mechanoreceptors [20].

**Figure 1.**
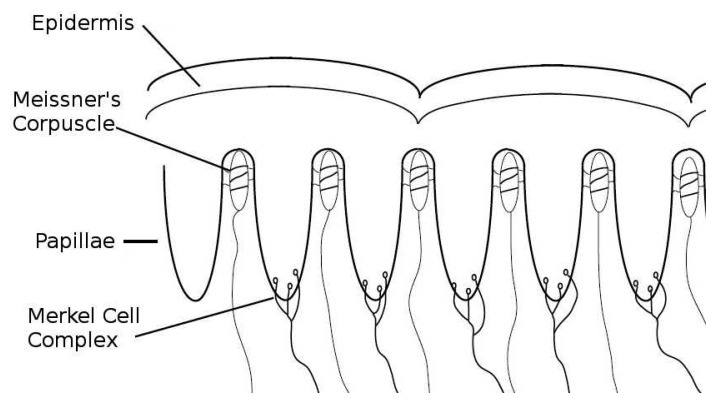
Cross section of human skin, showing the approximate locations of the different mechanoreceptors.

The tactile fingertip (TACTIP) sensor used in this study is biologically-inspired, taking inspiration from the mechanisms and multi-layered structure of human skin [14,15]. The TACTIP exploits recent theories about how the papillae structures (intermediate epidermal ridges) on the underside of the epidermis interact with the Meissner's corpuscle receptors to provide highly sensitive encoding of edge information [21,22]. It is suggested that changes in the surface gradient of the skin due to tactile interactions create deflection patterns of the papillae, which activate the Meissner's corpuscles that lie between them [14]. The presence of the papillae may also lead to higher stresses near the Merkel cells, positioned at the tip of each papilla [23]. Figure 1 shows a cross section of the human glabrous skin, which illustrates the papillae structures and placement of the mechanoreceptors. According to studies that focus on human and monkey skin [22,24], the frequency response of Meissner's mechanoreceptors is approximately 8–64 Hz [25,26], with a receptive field of 3–5 mm [27] and a sensitivity to indentation that begins to saturate beyond around 100 *μ*m [28]. Merkel mechanoreceptors operate at lower frequencies of 2–32 Hz [26], can resolve smaller spatial details, and are able to encode skin indentation beyond 1,500 *μ*m [27,28].

**Figure 2.**
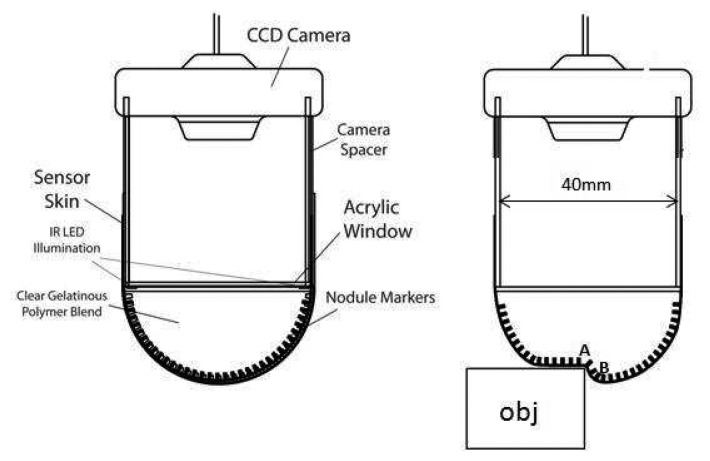
Structure of the biologically-inspired vision-based tactile sensor, tactile fingertip (TACTIP) (**left**). Interactions with a rigid object (**right**) causes localized papillae deflection at points A (divergence) and B (convergence).

The sensor replicates the papillae structures in the human skin using an array of short pin-like nodules on the underside of its skin-like membrane. Figure 2 shows the sensor architecture and illustrates the sensor concept, where the papillae are deflected as the result of surface deformation. The opaque skin-like membrane consists of a 40 mm diameter hemisphere of 0.3mm thick, black, Shore hardness A 50 urethane, which provides a flexible but strong and relatively inelastic layer. The array of papillae-like nodules is moulded onto the internal surface of this skin layer, with the tips colored white to aid localization on the black membrane background. This epidermal surface encloses a clear, highly compliant polymer that mimics the dermis and subcutaneous fat in the human finger whilst allowing the underside of the membrane to be viewed through a camera. The artificial skin layers have similar mechanical responses to indentation and shear as the human finger pad but they do not exhibit as much hysteresis. A more non-elastic sensor filling could be attractive for providing greater skin curvature and therefore papilla deflection during interactions, especially with soft elastic objects, although that is not the focus of this performance evaluation. When an object interacts with the sensing surface, changes in the surface gradient of the sensor membrane cause displacement of the white papillae tips on the underside. A CCD camera is used to capture the positions of the white papillae tips. The camera is mounted at a distance of approximately 50 mm from the centre of the membrane in order to capture the whole marker array with almost uniform focus. Six infrared LEDs are positioned above the papillae array to illuminate it. The spatial resolution of the sensor for tactile information relies on the papillae density and the image capture and processing system.

The main advantage of this optical approach to tactile sensing is the removal of any sensing elements or electronics from the immediate locality of the sensing surface. This means that a high spatial resolution can be attained without affecting the softness of the surface. Furthermore, the sensing surface is also very durable, with significant protection between the environment and any delicate components. The resultant device is suitable both for manipulating and for feeling objects, just like the human finger.

### 1.2. Feature Extraction

**Figure 3.**
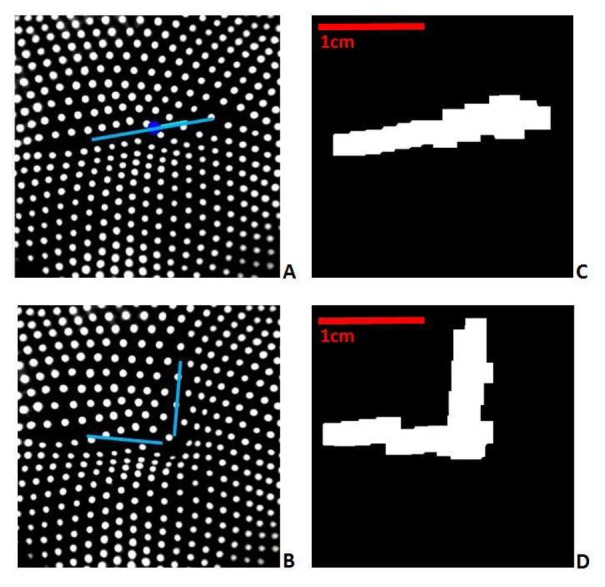
Two examples of surface features identification; (**A,B**) embedded camera view, (**C,D**) extracted edge features.

Figure 3A,B show typical papillae distributions as captured by the embedded camera. In this study, tactile features are extracted by detecting the area and direction of surface gradient changes. Two morphological image processing operators are used to detect these gradient features directly from the papillae marker images, thereby avoiding more processing intensive methods such as tracking each individual marker. The relative displacement of papilla groups are mapped through real-time local aggregation operations to higher level features including lines and points, and dynamic responses such as force and shear. This method is very quick and forms the initial image to be passed to the control software. The operation of image processing functions used in the algorithms presented (Dilation and Erosion) generate the output as result of the contribution of black and white (inhibition, excitation respectively) neighbors, mimicking the aggregation of local information in biological neurons.

We first pre-process the camera image to reduce noise and perform contrast and light adjustments. This source image is then dilated (the bright regions are expanded) *n* times using kernel *K*_1_. The higher the number of iterations, the greater the effect the function has on the image. In this work, *n* = 5. The value was determined experimentally by considering the papilla spot dimension on the image, the distance between them, and the total image size. This dilation enlarges the white spots to such an extent that close markers merge together. Following this action, the image is eroded (the bright regions are isolated and shrunk) 2*n* times using kernel *K*_2_. Finally, the image is cleaned by applying a binary threshold function to obtain a black and white image. In these experiments the default kernels were implemented in the OpenCV framework as 3 × 3 matrices. In this study we select *K*_1_ = *K*_2_ for simplicity. Pseudo-code for this algorithm is shown below.

**Algorithm 1**
1: Frame Process2: Dilate Function (cvDilate(*I*, *n*))
$\text{Dilate}(x,y)=\frac{\mathrm{Max}}{(x^{\prime },y^{\prime })\in K_{1}}I(x+x^{\prime },y+y^{\prime })$3: Erode Function (cvErode(*I*, *n*)) 
$\text{Erode}(x,y)=\frac{\mathrm{Min}}{(x^{\prime },y^{\prime })\in K_{2}}I(x+x^{\prime },y+y^{\prime })$4: Clean5: End


The result of this processing can been seen in Figure 3. This figure illustrates the extraction of features from the embedded camera sensor, where an edge is detected from the deflection of the internal papillae. Figure 3A,B show the camera view of the papillae ends when a horizontal edge and a corner are sensed by the fingertip. The lines show the position and orientation of the actual edges. Figure 3C,D show the corresponding features extracted using the method described above. Note the clear correlation between the extracted shapes on the right and the lines on the left.

Executing the tactile feature extraction algorithm on a single-core 3 GHz processor computer takes 16–30 ms each frame, depending on the thread scheduling. This results in an upper-bound of approximately 60 frames per second (fps), which covers almost the entire bandwidth of the Meissner's corpuscle. However, whilst 60 Hz or even higher frequencies are possible in terms of sensor bandwidth [17], the processing was restricted to 25 fps in this work due to the maximum capture rate of the camera.

After processing, the resulting frames, as illustrated in Figure 3C,D, contain all the information needed for subsequent feature detection. The white region represents the changing gradient of the target surface. The final step of the processing then involves extracting higher level information from this binary image.

For the contour-following tasks used in this study, the extracted features are subsequently processed in order to obtain information suitable for the control software. For instance, the orientation of a detected edge is processed using the principle and secondary Eigen components of the region (*i.e.*, the image moments [29]).

## 2. Experiments in Real-time Active Touch

The aim of the experiments was to quantitatively define the capability of the sensor for human-like environmental exploration and shape recognition tasks, and to compare this capability to that of humans conducting a similar tactile exploration task.

To achieve this, the sensor was mounted on a robotic arm to allow it to be moved through the environment and explore object surfaces and edges. The sensor position was controlled autonomously according to real-time processing of the tactile sensor information.

**Figure 4.**
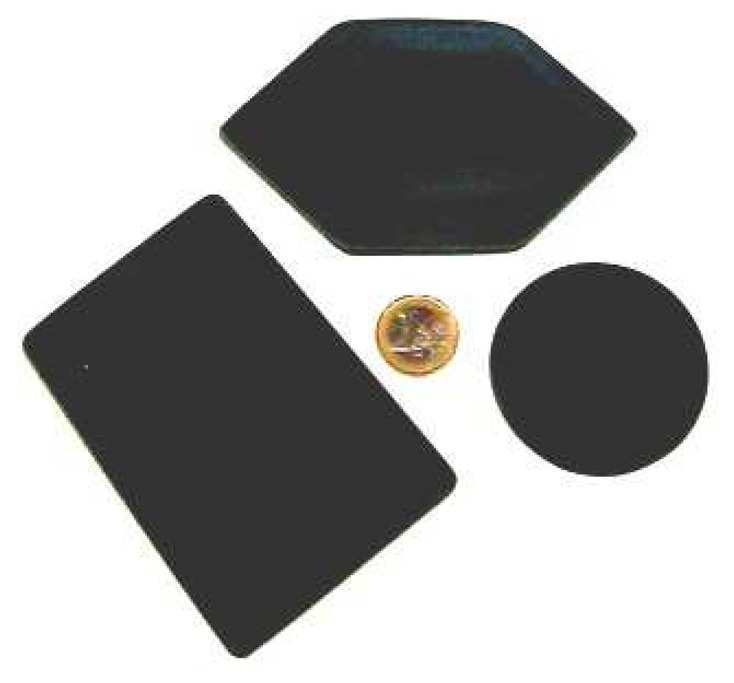
The target objects used in the experiments constructed from 3 mm thick polycarbonate.

A number of raised shapes (shown in Figure 4) were presented to the system and the paths taken by the sensor when following the perimeter edge of the shape were recorded. The shapes were constructed from 3 mm thick rigid plastic, even though the sensor can detect different thickness objects below 1 mm using the current hardware and software implementations.

Volunteer Human test participants were also asked to feel around the edge of these same shapes. The paths taken by the sensor system and the human subjects were compared to each other, and to the actual object shapes and sizes of the objects. This information was used to analyze the performance of the sensor system.

### 2.1. Robotic Contour Following Task

The main hardware components of the setup were:

1. Robotic platform to control the sensor position
2. Target shapes
3. Cradles to secure the sensor on to the platform

**Figure 5.**
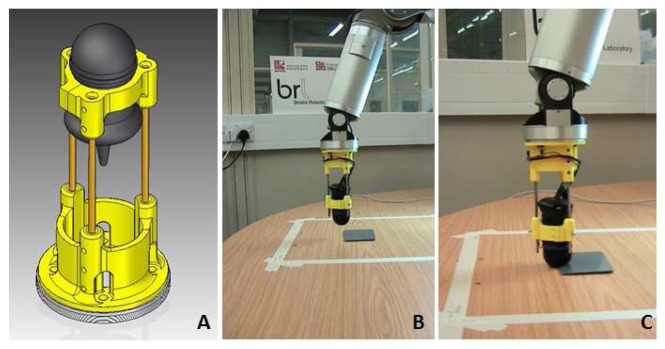
(**A**) sensor mounted in its cradle, (**B**) the cradle mounted on the WAM (Whole Arm Manipulator) approaching the environment and (**C**) the fingertip on the target during the active touch task.

A Barrett Technology Inc, 7DOF robotic arm was used for the robotic platform to move the sensor (shown in Figure 5). The platform operates within a defined working area of 400 *mm*×400 *mm* square, in roughly 1 mm increments.

No information from the robot arm positioning system was used (such as absolute position, force, or velocity). This is somewhat different to human tactile exploration where a degree of proprioception and visual feedback normally accompanies tactile exploration. However, this choice provides a more stringent evaluation of the fingertip sensor.

**Figure 6.**
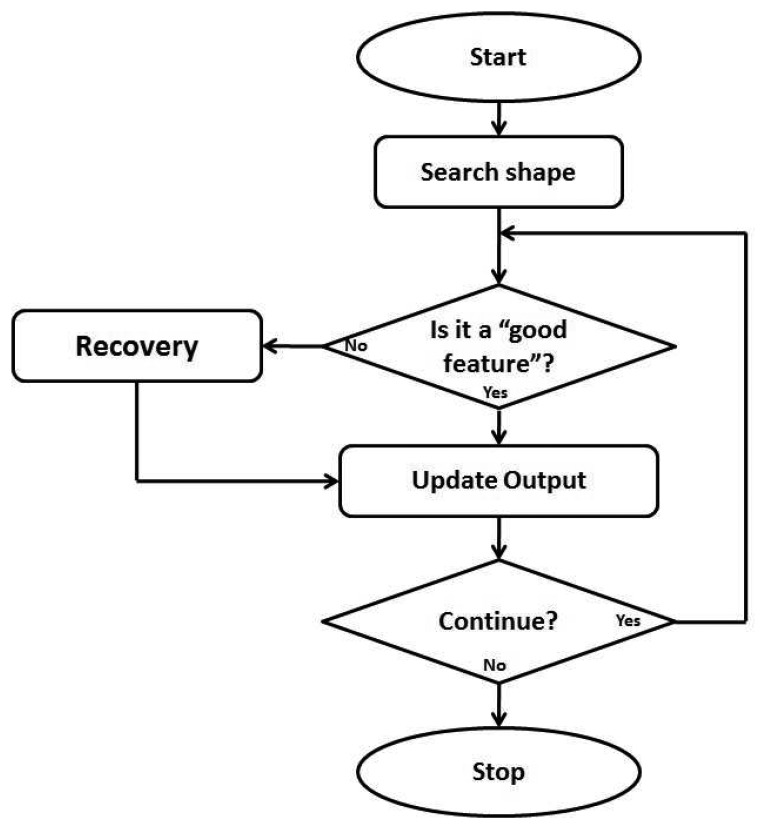
The decision process used by the algorithm. At the beginning, the software searches for an edge by moving using the robotic arm. When the sensor starts to detect a possible edge, the shape found event is triggered in order to start the contour-following task. Once an edge is found, the gradient is determined and used to test for a ‘good feature’. If this is satisfied the position and details are recorded and the next movement calculated. When the starting point is again reached, a stop command is triggered.

The exploration process for the sensor is described in Figure 6. By applying the low level image-based feature extraction algorithm detailed earlier, the edge angle and edge orientation features were extracted from each image frame. By edge angle we mean the direction of the edge on the table plane and by edge orientation we refer to the side of the edge which is highest (in order to define the inside and outside of the object).

This information was then used to determine the direction of movement for the sensor, with a consistent movement direction chosen according to the edge orientation. Changes of the direction of motion as the edge were quantized to 45 degrees to simplify the output evaluation. This choice affects the accuracy of the output but the effect is small due to the small 1 mm step size of the positioning system.

**Figure 7.**
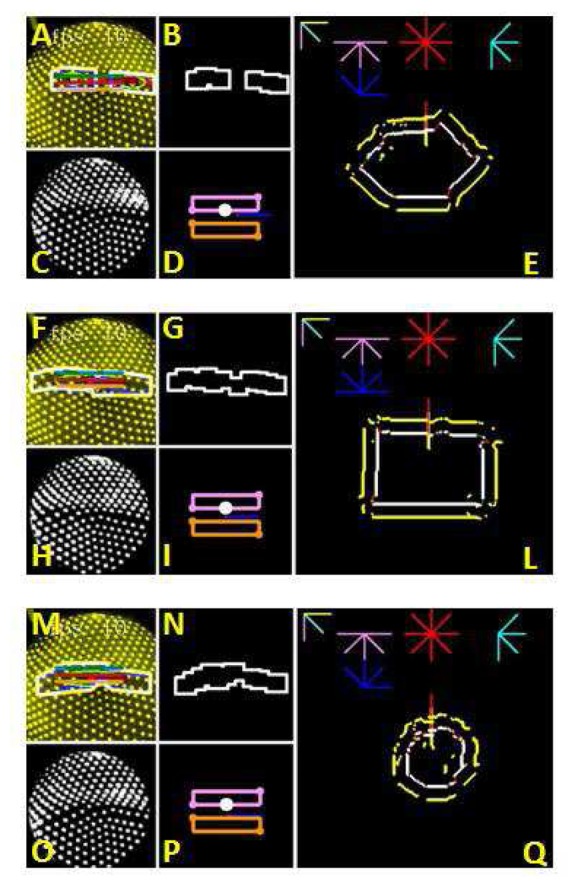
The various processing steps and outputs as the sensor system finds and explores each test object. The largest images show the early reconstruction stage, using the whole path. **C,H,O** show the live video from the camera. **A,F,M** show both the sensor image and the edge detection algorithm output. **B,G,N** show the extracted feature from the algorithm alone. Finally, **D,I,P** show two equal areas parallel to the detected edge. Within each of these areas, the number of papillae is calculated so the higher side of the edge can be found (due to the convergence or divergence of the papillae).

Figure 7 shows examples of the main processing steps of the proposed algorithm (small images) and the 400×400 pixel output reconstruction (large images). The larger images show the reconstructed shape formed using the sensor data and the calculated sensor position. When an object is encountered (when the sensor output detects an edge), the tactile features of the edge are assessed with respect to a notion of whether it is a ‘good feature’. A frame contains a ‘good feature’ if: (i) a feature is present, and (ii) the extracted feature's centre of mass is within the central zone of the camera. This zone is about 1/3 of the image size. This check ensures that the sensor is on the edge of the shape before extracting the possible direction for the next iteration. The frames without a ‘good feature’ contain however information for the recovery process that will move the sensor in the direction of the centre of mass. This operation leads to a new ‘good feature’ and the process can start once again. This notion is similar to the capabilities of humans to first explore the macro scale properties of an object (the size and general shape) before refining this to determine the minor scale properties. To this extent the object exploration algorithm first seeks a ‘good feature’ upon which it may base further active sensing. The higher side and the falling side of the edge are calculated by defining two rectangular regions (visible in Figure 7D,I,P and within these regions, detecting the amount of white (papillae) in the image; the greater the white value, the greater the number of papillae. Due to the aggregation of papillae in the higher edge and divergence in the falling side, which is an intrinsic feature of the sensor, the edge orientation can be detected.

A detected edge is classified as a ‘good feature’ when the desired tactile features (angle and orientation) can be found and the centre of the edge is positioned in the middle of the sensor. Where a ‘good feature’ is not found, the sensor must be moved to try to recover the edge. This method gives two main advantages: First, the centre of the feature is always coincident with the sensor's most sensitive area and second, any horizontal displacement of the soft sensitive surface due to shear forces corrected. Without this correction, this latter point can result in a mismatch between the centre of the image and the centre of the sensor due to the friction between the surface and the sensor membrane causing a displacement of the compliant sensor surface.

Theoretically, the algorithms used can run with an update rate of 60 fps and therefore the speed of the fingertip could be potentially increased. In this work, in which maximizing the speed is not a primary objective, the frame and the update rate are determined by the 30 fps maximum camera refresh rate and the speed of the arm, limited to 10 mm/s in order to minimize the risk of damaging the sensor skin whilst sliding along the objects' edges. This velocity was deemed to provide a reasonable trade-off between speed of active exploration and reconstruction fidelity.

### 2.2. Human Contour Following Task

As a bio-inspired sensor, the TACTIP sensor is not optimized for precision, unlike a digital sensor such as a laser scan array, but rather is optimized for a balance between compliance, robustness and accuracy. However, even the human tactile perception is not 100% precise. Consequently, a set of experiments has been designed in order to evaluate the sensor in terms of the performance of the human sense of touch. By comparing the results of the artificial tests and the human ones we can define whether the sensor can effectively mimic human touch. The human experiments were designed not from a neuroscientific point of view but as an engineering tool to obtain data for a quantitative comparison.

Twelve volunteer subjects were asked to perform object exploration and identification tasks. Each subject was blindfolded throughout the tests and was unfamiliar with the objects. The dominant hand was used and the subject was instructed to not move the index finger during the experiment independently from the hand.

The experiments were divided into three tasks in which the rectangle, hexagon and circle were presented to the volunteers. The volunteer was free to explore the shapes by touch alone using one fingertip. They were then required to guess the kind of shape and to estimate its dimensions as feedback about the subject's mental reconstruction in a short interview.

**Figure 8.**
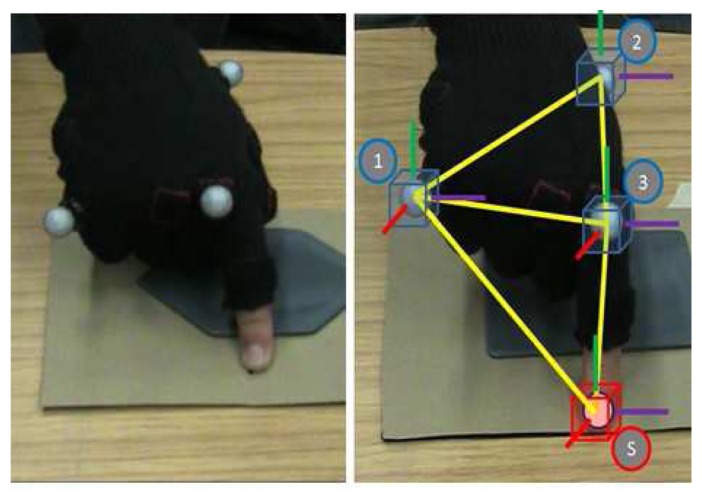
The left picture illustrates the sensorised glove with the Vicon markers. The index finger glove tissue was removed to allow full skin contact with the surface. In the right image, the tracking point and frame of reference for fingertip location are highlighted. 1,2,3 are the real, tracked markers. ‘S’ is the virtual marker. The position of ‘S’ is recorded.

No constraints were imposed on the exploration speed. To generate the human fingertip trajectory during these tests, they were preformed within a Vicon optical motion tracking environment. Through the use of small markers and Infrared cameras, the Vicon System can reconstruct the movements of a rigid body. In these experiments three markers were placed on a glove worn by the subjects and these formed a known rigid body within the Vicon workspace with the *reference point* fixed on the fingertip. The glove, marker and fingertip are illustrated in Figure 8.

The experimental constraints imposed during these tests were:

1. Subjects were blindfolded
2. Only one fingertip was used, from the index finger
3. The hand was held in as close to a constant orientation as possible and the index finger not moved independently from the hand
4. Movements were preferred in one direction (*i.e.*, following the circumference of an object)
5. After exploring the object, an estimate of the shape and its dimensions was made

## 3. Results

**Figure 9.**
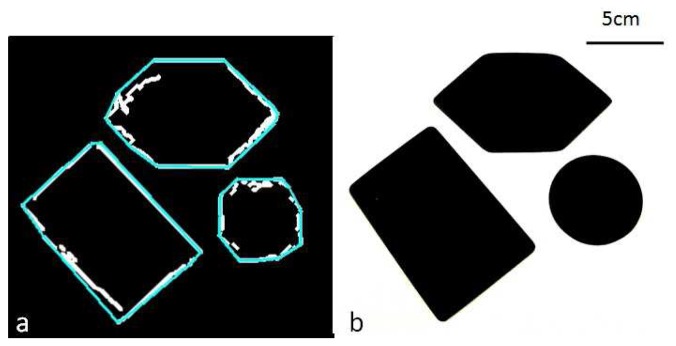
Visual comparison of the artificial sensor path (**a**) in white, reconstructed shape in blue and the target shapes (**b**).

As shown in Figure 7, the artificial fingertip and autonomous control system are able to successfully map the perimeter of the different objects by finding and then following their raised edges. A comparison between the real and estimated perimeters of the shapes by the sensor system can be seen in Figure 9. The trajectories are not always completely closed due to the stop condition, although this is not a problem for the evaluation due to the post process convex hull built around the trajectory (Figure 9a). The length of the hull sides was used to generate the reconstructed dimensions.

Table 1 compares the dimensions of the reconstructed shapes found from the sensor's path to the actual dimensions of each shape. It is clear that the dimensions of the paths taken by the sensor are very similar to those of the actual objects.

**Figure 10.**
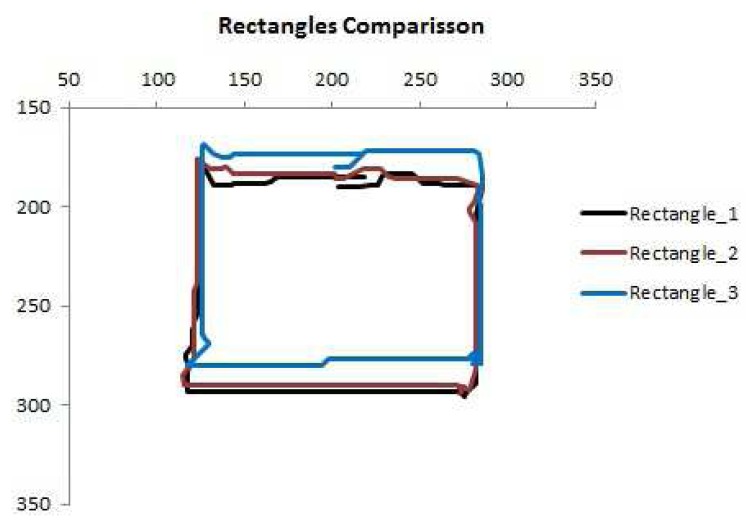
Three reconstructed rectangles using the sensor paths.

The repeatability of the reconstructed rectangles is high and can be observed in Figure 10, which shows an example of three different exploration paths, overlapped, for the rectangular object. Comparison of the side lengths in each case shows that they are equal with an accuracy of 3%. The maximum estimation error for the area of these paths is calculated to be approximately 7.5%.

**Figure 11.**
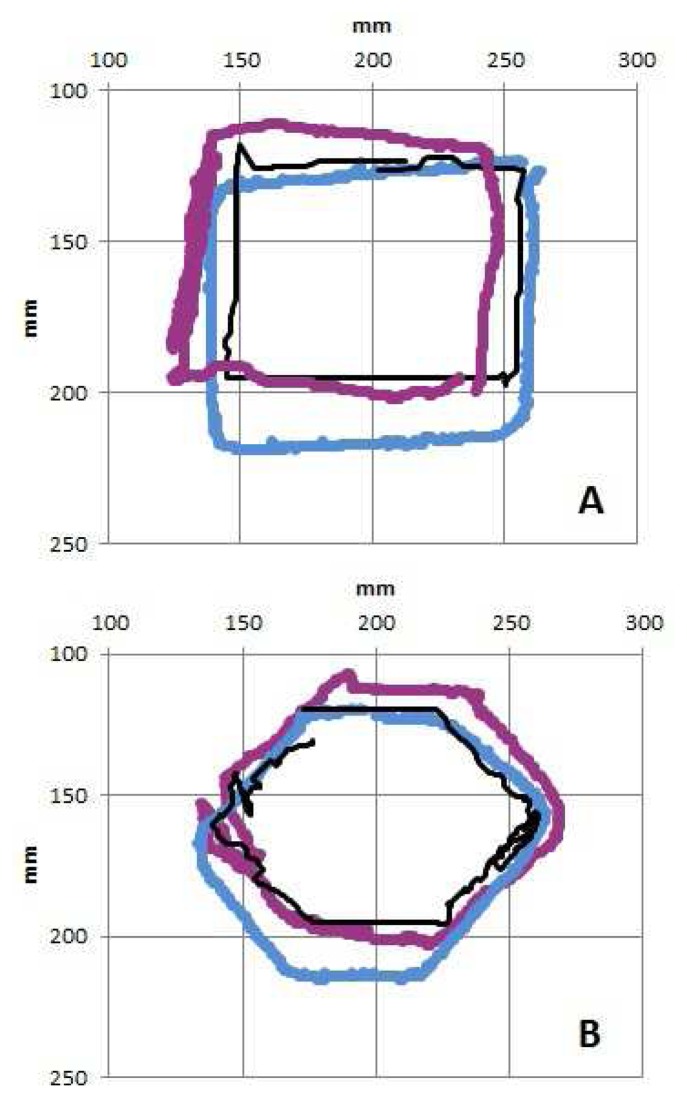
Comparison of the shape reconstructions between two humans and the sensor system for the rectangle (**A**) and the hexagon (**B**). The thinner black lines are the sensor trajectories.

Comparison of the human trajectories derived from the Vicon tracking data with those of the sensor shows great similarity (Figure 11). Two human trajectories and one from the robotic platform are shown in this figure. It is not immediately clear which is the artificial one.

**Figure 12.**
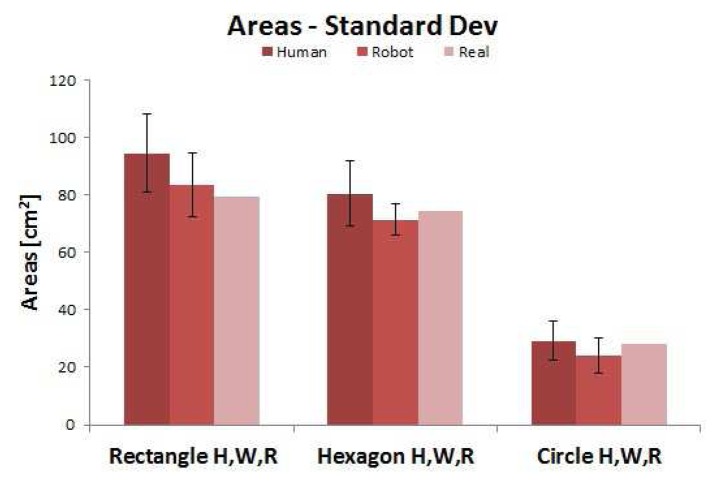
Comparison of mean estimated areas for humans, robotic platform and real dimension.

Analysis of the mean object areas for 12 human subjects and the comparison with the robotic system, shown in Table 2, indicates that there is an approximately constant scaling factor ( $\left(\bar{A_{H}}\right)/\left(\bar{A_{P}}\right)$) between the two of approximately 1:1.1, with the human paths enclosing a slightly larger area. Figure 12 compares this data to the actual object areas. The scaling factor is again evident, as well as the similarities in performance between the biological and artificial systems being highlighted once more.

## 4. Discussion

The tactile processing algorithm was able to reliably detect the raised object edges through the artificial fingertip, with minimal adjustment for the object height, dimensions or other specific attributes. It is therefore expected that this system could be used for many different tactile applications, especially given the diverse tactile information offered by the novel TACTIP device.

The autonomous sensing system, created by mounting the TACTIP on a robot arm and moving it according to the tactile data, was also found to be very reliable given the naive positioning used. The simple positioning algorithm used the tactile information from a single location to calculate the subsequent movement direction. A future revision will use a selection of previous tactile data points to determine a better candidate trajectory. However, one limitation of the current algorithm is the recovery mechanism. In this work, if the fingertip moves away from an edge recovery has been simply addressed by slowly moving back to the previous ‘good feature’ position until new ‘good features’ are detected. In the few cases were this strategy was not enough to achieve recovery, the edge was lost. To improve this, the recovery mode could be extended to include a circular palpation pattern, or its path could be retraced, until a ‘good feature’ is regained. Such a procedure was not implemented during these experiments. The focus here is on tactile feedback alone, with no direct feedback of the sensor's position.

The object perimeters found by the artificial tactile system compare well with those by the human subjects. The scaling factor between the two, where the human paths were approximately 10% larger than the sensor system's, is expected to be due to the human subjects using a kinesthetic element to aid the navigation, by applying a slight inward force against the objects' outside edges. In doing this, the centre of the finger would be positioned on the outside of the edge rather than directly above it. A similar mechanism could be applied to the artificial sensing system using force feedback as measured by the TACTIP fingertip sensor.

## 5. Conclusion

A novel biologically-inspired tactile system using the TACTIP compliant sensor was designed and shown to complete real-time object exploration tasks in a structured environment with a human-like performance. The sensor utilizes the movement of papilla-like structures on the underside of its artificial epidermal layer to detect changes of the sensing surface. This is a similar mechanism to that used in human skin. An optical sensing method is used to detect the papillae deflections, which avoids placing delicate sensing elements near the skin surface and leads to a very robust and compliant device. The proposed algorithm avoids computationally expensive tracking methods by applying fast image operations to the sensor output to extract tactile features.

The TACTIP device was mounted on a robotic arm in order for the system to feel and follow the edges of raised objects. The edge angle and orientation at each sensor position was used to determine a movement direction, following which the new tactile data was analyzed. The object perimeters found by the autonomous system were compared to those found by human subjects during similar tasks. The results for this comparison show high similarity, and that the sensor can identify and parameterize hard edges effectively using the proposed algorithms. This shows that the artificial fingertip sensor is capable of emulating human tactile sensing performances despite the human subjects exploiting a larger amount of information not provided to the sensor elaboration such as proprioception, force feedback, vibration, temperature, and texture.

The sensing solution is both sensitive and robust. These attributes make it suitable for tactile sensing as well as gripping and manipulating objects. It is therefore expected to provide a good solution for active sensing and environmental exploration, whilst offering a performance similar to that of humans.

Useful future work could investigate the sensor's ability to detect and follow the edges of different 3D or compliant objects compared to that of humans. The benefit of detecting and processing information about the depth of indentation would also be useful to determine the benefit of changing the current algorithm to include this additional dimension.

## Figures and Tables

**Table 1.** The reconstructed shapes' dimensions compared to the real ones, for the sensor system. [Dimensions in mm], (real dimensions in brackets).

Sides #	Rectangle (real)	Hexagon (real)	Circle (real)
1	10.4 (10.8)	4.8 (5.4)	-
2	7.0 (7.5)	5.0 (5.3)	-
3	10.4 (10.7)	4.9 (5.3)	-
4	6.6 (7)	5.1 (5.4)	-
5	-	4.8 (5.3)	-
6	-	4.6 (5.3)	-
Diam.	-	-	6.1 (6)

**Table 2.** Mean areas and the ratio of the areas circumscribed by the humans $\left(\bar{A_{H}}\right)$ and the robotic platform $\left(\bar{A_{P}}\right)$.

-	$\bar{A_{H}}$ [*cm*^2^]	$\bar{A_{P}}$ [*cm*^2^]	$\bar{A_{H}}/\bar{A_{P}}$
Rectangle	94.540	83.570	1.13
Hexagon	80.473	71.333	1.13
Circle	29.250	27	1.08
